# Sternal Complications Following Coronary Artery Bypass Grafting and Robicsek Repair: Comprehensive Sternal Reconstruction With Sternal Plating and the Use of Novel Biologic Therapies

**DOI:** 10.7759/cureus.59719

**Published:** 2024-05-06

**Authors:** Ujjawal Kumar, Usman Aslam, Zain Khalpey

**Affiliations:** 1 Department of Cardiothoracic Surgery, HonorHealth, Scottsdale, USA; 2 School of Clinical Medicine, University of Cambridge, Cambridge, GBR; 3 Department of General Surgery, HonorHealth, Phoenix, USA

**Keywords:** sternal plating, coronary artery bypass graft surgery, fractured sternal wires, sternal non-union, robicsek repair, sternal wound complications

## Abstract

Sternal non-union and fractured sternal wires are rare but devastating complications of median sternotomy for cardiac surgery, and these can lead to chronic pain, instability, and impaired quality of life. Patients may present with various symptoms such as clicking sensations, chest wall discomfort, and even respiratory difficulties. The underlying causes are multifactorial, including patient comorbidities, surgical technique, and postoperative management. The treatment options range from conservative measures to complex surgical interventions, such as sternal debridement, rewiring, and reconstruction with rigid fixation systems. Novel therapeutic technologies, including amniotic membranes and platelet-rich plasma, have shown promise in promoting wound healing and reducing complications in these challenging cases.

We present the case of a 58-year-old male who underwent coronary artery bypass grafting (CABG) and subsequently developed sternal dehiscence requiring Robicsek repair. Despite undergoing this procedure, the patient experienced poor sternal healing, and hence he was referred to our center, presenting with shortness of breath, pain due to fractured sternal wires, and sternal non-union. The patient underwent a complex sternal reconstruction involving redo full median sternotomy, removal of sternal wires, and sternal plating, along with the application of amniotic membranes and platelet-rich plasma to the sternal wound. The procedure successfully stabilized the sternum. This report highlights the benefits of a multifaceted approach to addressing repeated sternal breakdown following CABG and the potential therapeutic benefits of novel technologies in promoting wound healing.

## Introduction

Despite the significant advances in minimally invasive approaches in cardiac surgery, the full median sternotomy remains the most common approach for surgical procedures of the heart and great vessels [1]. Healing complications following median sternotomy, including instability, non-union, infection, or dehiscence, are rare but can have devastating consequences with significant morbidity and increased mortality rates [2]. These complications also lead to prolonged hospital stays and high healthcare costs [3]. In the long term, they may result in chronic pain, chest wall instability, and impaired quality of life, often manifesting postoperatively with symptoms such as clicking sensations, chest wall discomfort, and respiratory difficulties [4,5].

The causes of sternal healing complications are multifactorial and may involve intrinsic patient characteristics and comorbidities, surgical technique, and postoperative management [2]. The risk factors include obesity, diabetes mellitus, chronic obstructive pulmonary disease (COPD), advanced age, and osteoporosis, as well as the use of bilateral internal mammary arteries (BIMA) for coronary artery bypass grafting (CABG) [6]. Obesity and diabetes pose significant risks due to their association with poor wound healing and increased infection risk [7]. COPD and significant smoking history, as well as advanced age, contribute to sternal instability due to chronic coughing and weakened bone structure respectively [8]. While using BIMA for grafting can provide excellent long-term outcomes for graft patency overall, it may compromise sternal blood supply, thereby leading to impaired sternal healing and an increased risk of dehiscence in some cases [9].

Managing sternal dehiscence is complex and depends on the severity of the condition, the presence of infection, and other factors. Conservative treatments like wound care and vacuum-assisted closure therapy may suffice for superficial dehiscence without infection [10]. However, in cases of deep sternal wound infection (DSWI) or severe sternal instability, surgical interventions such as sternal debridement, rewiring, and reconstruction with rigid fixation systems may be necessary [11]. The Robicsek repair technique involving sternal weave with stainless steel wires has been widely used to increase sternal stability and improve outcomes compared to conventional methods [12,13]. Recently, titanium sternal plates have also gained popularity for high-risk patients [14]. Novel therapeutic techniques like amniotic membranes and platelet-rich plasma have also shown promise in promoting wound healing [15,16].

We present the case of a 58-year-old male who underwent CABG and subsequently developed sternal dehiscence requiring Robicsek repair. Following primary repair, the patient once again experienced poor sternal healing and was referred to our center with shortness of breath, chest wall pain from fractured sternal wires, and sternal non-union. He underwent complex sternal reconstruction involving redo full median sternotomy, removal of sternal wires, and sternal plating in addition to the application of amniotic membrane and platelet-rich plasma to the wound. The procedure successfully stabilized the sternum. This report underscores the benefits of a multifaceted approach to addressing repeated sternal breakdown following CABG surgery and illustrates the potential therapeutic advantages of novel technologies for promoting wound healing in such cases. By emphasizing the importance of identifying high-risk patients and implementing appropriate preventive as well as treatment strategies, we aim to contribute further evidence on this topic while enhancing patient care in the cardiac surgery field.

## Case presentation

A 58-year-old male under the care of another hospital system presented to his cardiac surgeon’s office in January 2024 with a chief complaint of dyspnea. He had previously undergone four-vessel CABG surgery in May 2022, performed by the cardiac surgeon whose office he presented to.

Index admission: operative details

The left internal mammary artery had been used to bypass the left anterior descending artery (LIMA-LAD), a saphenous vein graft was used sequentially to bypass a diagonal branch of the LAD and an obtuse marginal branch of the left circumflex artery (SVG-Diag/OM) and another saphenous vein graft was used to bypass the right posterior descending artery (SVG-rDPA). Other than significant four-vessel coronary artery disease that necessitated surgical revascularization, his medical history was significant for poorly controlled type 2 diabetes mellitus and hypertension as well as well-controlled hyperlipidemia. He was undergoing treatment for his type 2 diabetes with weekly subcutaneous injections of dulaglutide, a GLP-1 receptor agonist, as well as injections of insulin daily before meals. For his hypertension, he was receiving treatment with losartan, atenolol, and carvedilol. He had a BMI of 38.1 kg/m^2^. He was a former smoker with a 20-pack-year smoking history but had quit smoking before his CABG surgery; he had adequate pulmonary function on spirometry.

Before the CABG surgery, the patient's preoperative echocardiogram had shown normal left ventricular size. The left ventricular ejection fraction had been mildly reduced at 50-55%, with regional wall motion abnormalities noted, particularly in the anterior and inferior walls, suggestive of ischemia or prior myocardial infarction in the territories supplied by the coronary arteries requiring bypass grafting. No significant valvular abnormalities had been observed. The right ventricular systolic and right atrial pressure had been within normal limits, at 28 mmHg and 4 mmHg, respectively. No pericardial effusion had been detected on echocardiography.

His postoperative recovery following the CABG surgery had been complicated by recurrent pleural effusions, which required six thoracentesis procedures, as well as exploratory video-assisted thoracoscopic surgery (VATS). Thoracentesis had shown an excess of serous fluid, which was drained. On analysis, a predominantly raised level of neutrophils had been seen, with S. pyogenes grown on culture. The ratio of protein in the fluid compared to the serum had been 0.62 and the ratio of LDH in the pleural fluid compared to the serum had been 0.71. These findings indicated an exudative process, likely due to infection and/or inflammation. Exploratory VATS findings had been largely unremarkable, with small, localized collections of fluid being seen, and mild pericardial and pleural inflammation visualized.

Additionally, the patient had reported experiencing significant dyspnea despite these procedures to relieve the recurrent pleural effusions. He had also reported chest wall instability and pain, and CT imaging had shown sternal dehiscence. He had therefore been taken back to the operating room for surgical reintervention, where the surgeon who had performed the original CABG procedure subsequently performed a sternal closure using running wires in a weave pattern as described by Francis Robicsek [12]. Following this reintervention, the postoperative recovery had been uneventful, and in mid-June, the patient was discharged home in a stable condition to continue recovery after a hospital admission of nearly a month.

Postoperative recovery after initial hospital admission

Following discharge, the patient's postoperative recovery at home had been initially without any complications. He had diligently engaged with a cardiac and physical rehabilitation program as instructed. However, during a follow-up appointment one year postoperatively, he reported occasionally hearing a "click" from his chest wall. He had also reported experiencing pain when coughing, prompting concerns for a degree of sternal breakdown with possible fractured wires. Given the patient's history of recurrent pleural effusions and his previous sternal repair, the patient was referred to our center for specialist evaluation and potential surgical intervention for complex sternal reconstruction.

Readmission: assessment by our specialist cardiac surgical team

**Figure 1:**
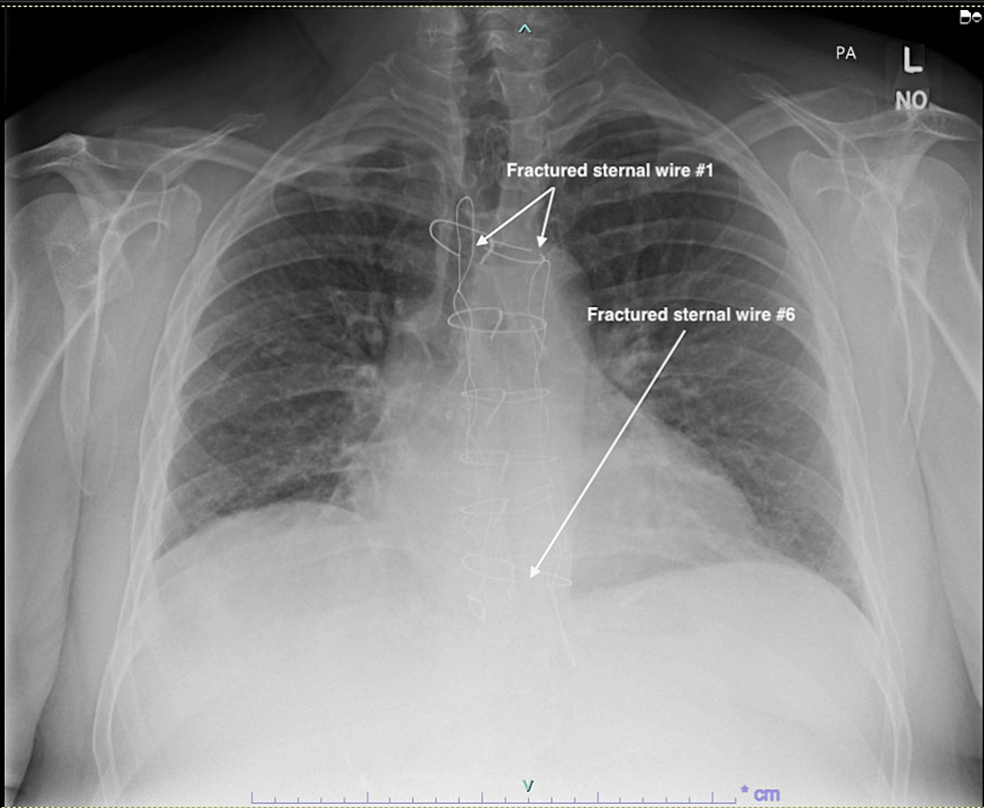
Preoperative chest X-ray image showing the fractured first and sixth sternal wires This postero-anterior (PA) chest X-ray was taken to investigate the symptoms that the patient was experiencing. It clearly shows the first and sixth sternal wires to be fractured. A slight tracheal deviation to the right side is also seen, though this could not be explained and was deemed to be an insignificant finding. The right and left Robicsek wires are seen and appear unremarkable

**Figure 2:**
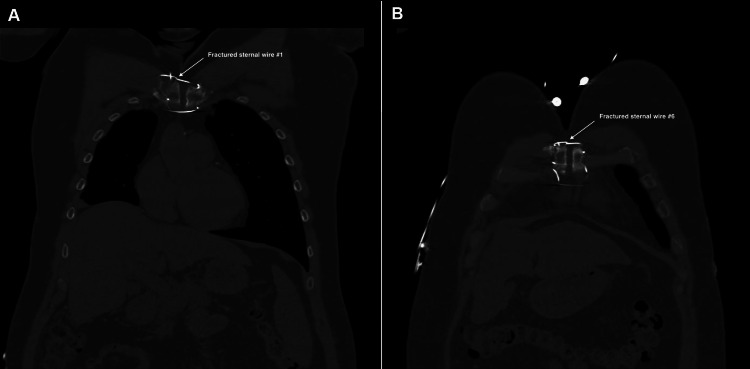
Preoperative CT imaging of the chest (coronal section) showing chronic sternal non-union as well as fractured first and sixth sternal wires The image shows a chronic non-union of the sternum, as well as fractured wire number 1 (panel A) and wire number 6 (panel B) CT: computed tomography

Before assessment in our outpatient clinic, the patient underwent a chest X-ray, as well as CT and echocardiography imaging for structural and functional evaluation. X-ray and CT chest imaging (Figures 1-2) revealed probable chronic non-union of the sternum with no evidence of dense bony bridging across the sternum. There was no evidence of any acute or concerning abnormalities in the anterior mediastinum or definite acute abnormalities in the chest. The imaging also showed that wires number one (Figure 2A) and six (Figure 2B) were broken.

Video 1 shows a transverse view of the chest CT imaging, demonstrating the full-length chronic sternal non-union, as well as the fractured wires one and six.

**Video 1:** Transverse section CT chest showing the chronic sternal non-union as well as the fractured sternal wires CT: computed tomography

The patient's preoperative echocardiogram showed normal left ventricular size and function, moderate concentric left ventricular hypertrophy, a preserved ejection fraction of 70%, and no significant valvular abnormalities. No wall motion abnormalities were noted, and there was deemed to be preserved systolic function, with a normal right ventricular systolic pressure of 26 mmHg, and a normal right atrial pressure of 3 mmHg. No pericardial effusion was seen on echocardiography.

The patient then underwent a comprehensive clinical assessment in the outpatient clinic at our center. It was determined that the sternal non-union and broken wires were causing significant chest wall instability and discomfort, contributing to the patient's dyspnea and overall decreased quality of life. The “clicking” was deemed to be due to the sternal non-union, and the pain on coughing was largely attributed to the broken wires “flicking” under the skin and subcutaneous tissues, causing pain. Given his history of recurrent pleural effusions, it was judged imperative to address the sternal non-union and chest wall instability to prevent further complications and improve his respiratory function. The patient’s CHA_2_DS_2_-VASc score was 2, the HAS-BLED score was 2, and the Thakar score was 0.4%. Considering the complexity of the case and the patient's high-risk profile due to underlying medical conditions, the decision was made to proceed with a complex sternal reconstruction.

Readmission: operative details of complex sternal reconstruction

Before the procedure, a multidisciplinary team pre-briefing led by the attending surgeon was held to discuss the patient's pathology and the indications for the surgery. The team reviewed relevant imaging findings from CT scans and echocardiography, as well as the specialized instrumentation and implants necessary for the procedure. The timing of key operative steps was also discussed to ensure a smooth and coordinated workflow. The patient was positioned supine on the operating table, and general endotracheal anesthesia was administered. The patient was prepped and draped in the usual sterile manner. A transesophageal echocardiography (TEE) probe was placed to monitor cardiac function throughout the procedure, and standard telemetry for cardiac surgery was set up. Gel pads were placed for cautery and cardioversion in case these interventions were required during the surgery. To monitor the patient's hemodynamic status, a right internal jugular (IJ) cordis and a Swan-Ganz central line were inserted. The Swan-Ganz catheter enabled continuous measurement of cardiac output and other flow dynamics parameters. Additionally, a right radial arterial line was placed for real-time blood pressure monitoring and arterial blood gas sampling.

**Figure 3:**
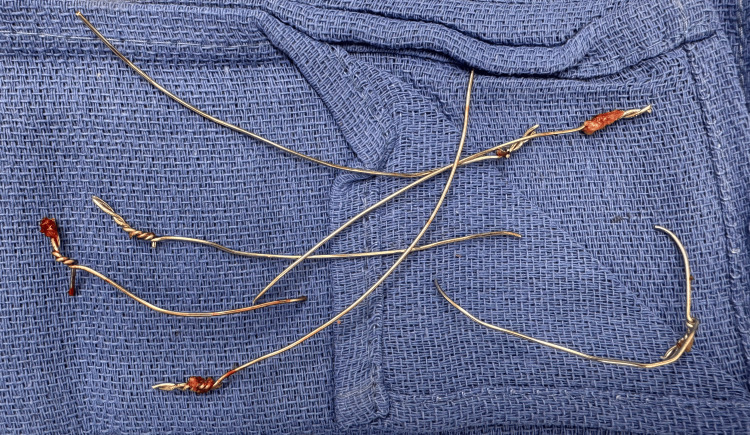
The original sternal wires from the initial procedure were removed during sternal reconstruction

Following a standard institutional timeout, the patient underwent a redo full median sternotomy to address a sternal non-union. Upon opening, the surgical team found no signs of infection but discovered the first and sixth sternal wires to be broken, and the left and right top Robicsek wires to be loose, as had been indicated by the preoperative CT scan. The sternum was also found to be unstable. Firstly, the original sternal wires were therefore removed (Figure 3).

**Figure 4:**
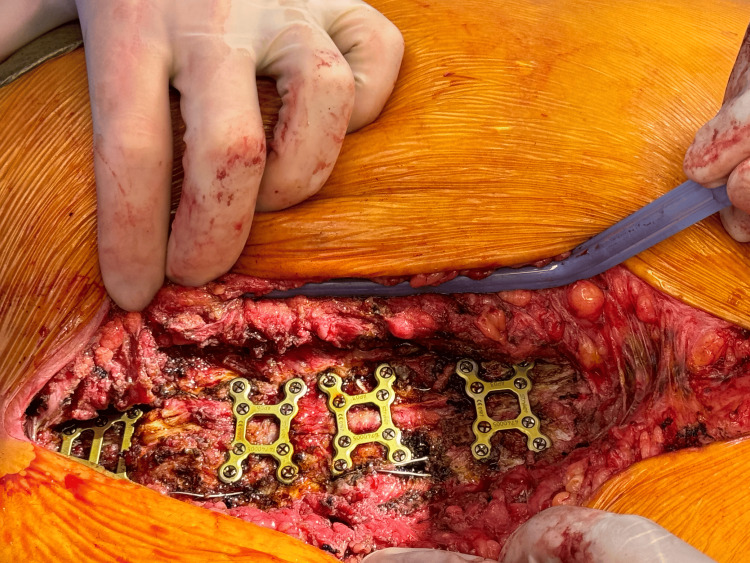
Stryker sternal plates (1 manubrial plate and 3 X-plates) and screws (4 x 10 mm and 16 x 12 mm) were used to stabilize the sternum postoperatively Three Stryker® X-plates were placed between the second and fourth, the fourth and sixth, and the sixth and eighth intercostal spaces. Additionally, a manubrial plate was also placed for extra stability. These plates were secured with four 10 mm screws, and sixteen 12 mm screws in total

Next, the two halves of the sternum were realigned and reapproximated, before the adjustment of the Robicsek wires. The right wire was tightened, and the left was partially moved. To stabilize the sternum, a manubrial plate and three X-plates (Stryker Inc., Portage, MI) were placed from the manubrium and between the second and fourth, the fourth and sixth, and the sixth and eighth interspaces along the sternum (Figure 4). These plates were secured using 10 mm screws (of which four were used) and 12 mm screws (of which 16 were used). The sternal edges were debrided and approximated, and a partial pectoralis flap was placed bilaterally to overlay the metal Stryker plates.

**Figure 5:**
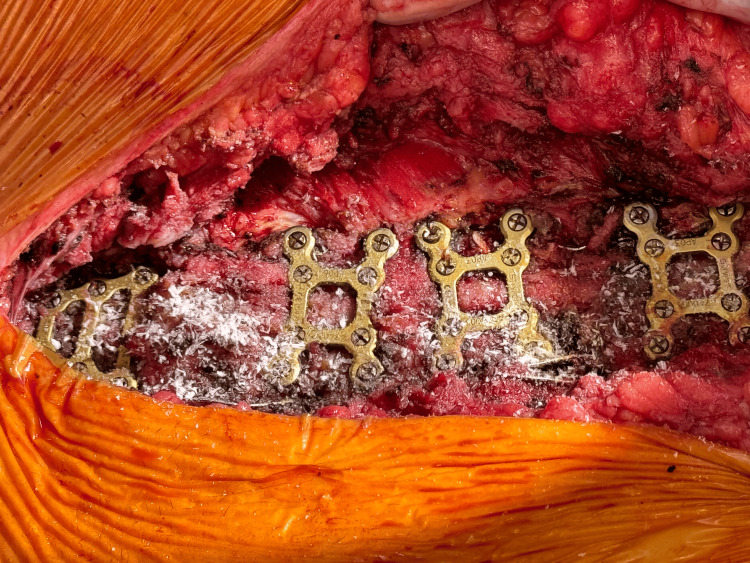
Salera® mini membrane was added to the wound to aid with wound healing

**Figure 6:**
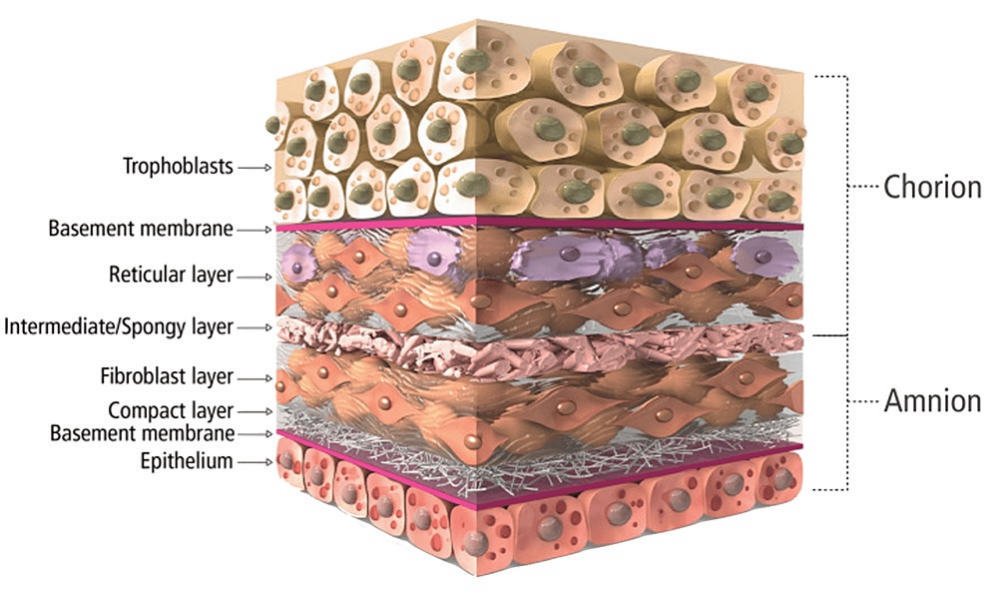
Cross-section schematic of an amnion-chorion placental allograft The figure used with permission from MTF Biologics

Before closure, 160 mg of Salera Mini Membrane (MTF Biologics, Edison, NJ) was placed over the sternum, and an additional 160 mg was used over the superficial fascia to aid in wound approximation. The area was washed and irrigated, and 2 x 160 mg of mini membrane amniotic material was applied (Figure 5). The amnion-chorion bilayer structure of the Salera® mini-membrane is shown in Figure 6.

Afterward, 120cc of pre-heparinized blood was spun down to obtain platelet-rich plasma, which was then applied to the wounds at the fascial level. The fascia and subcutaneous tissues over the sternum were closed in the usual fashion per our institutional practice. This was achieved in layers using 0 and 2-0 PDS Stratafix™ sutures (Ethicon Inc., Cincinnati, OH), and the skin was closed with one layer of subcuticular 4-0 Stratafix™ suture (Ethicon Inc.). Two Jumpstart FlexEFit Antimicrobial Composite wound dressings (Arthrex Inc., Naples, FL) were placed to cover the wound, and normal saline was added over its white strip for activation. The wound was closed without leaving a drain.

Preoperative and postoperative TEE findings and drips were effectively communicated between the anesthesiologist, OR staff, and the surgeon. The patient was transferred to the post-anesthesia care unit (PACU) for recovery, to be discharged home later that day. Postoperative analgesia was achieved using a minimal opioid strategy. To this end, the patient received a total of 90 mg of dronabinol (a synthetic cannabinoid) before and after surgery, with opioid dosage titrated to control pain. Dronabinol has been shown to reduce opioid analgesia requirements in cardiac surgical patients [17]. Postoperatively, blood sugar was controlled tightly within normal target ranges using a "sliding scale" variable rate intravenous insulin infusion.

Regular follow-up will be essential to monitor the stability of the sternum and assess the need for any additional interventions. As is standard in our institution, the patient will be seen in the outpatient follow-up clinic at two weeks, eight weeks, and six months postoperatively. Proper wound care and adherence to postoperative instructions will be crucial for optimal healing and recovery. The patient will also follow up with his diabetologist following discharge, who will review the blood glucose management regime to ensure better control in the future. The patient will follow a strict program of cardiac rehabilitation, which involves increasing amounts of cardiovascular and resistance/strength training to aid with weight loss. Additionally, the patient is expected to begin taking semaglutide (Ozempic), a GLP-1 receptor agonist that is increasingly used to achieve weight loss in obese patients, especially those with type 2 diabetes.

## Discussion

Sternal complications following median sternotomy for cardiac surgery, such as sternal non-union, and fractured sternal wires, can have devastating consequences for patients. This case report highlights the challenges in managing a patient with repeated sternal breakdown after CABG and Robicsek repair and the potential benefits of a multifaceted surgical approach incorporating sternal plating and novel biologic therapies. The causes of impaired sternal healing are multifactorial, including both patient and surgical factors [2]. In our case, the patient had several risk factors that likely contributed to poor sternal healing, such as obesity, diabetes, and significant smoking history [6,18]. Obesity and diabetes are known to impair wound healing and increase the risk of infection [7], elevating the risk of sternal wound breakdown. A significant smoking history can lead to frequent and forceful coughing, which was observed in our patient, increasing the risk of sternal instability postoperatively [8].

The exact causes of the recurrent pleural effusions that the patient experienced following the initial procedure were unclear and likely multifactorial. The patient suffered from postoperative hospital-acquired pneumonia and a surgical site infection. It likely progressed deep into the thorax, involving the pleura and the thoracic cavity, and developing into a deep sternal wound infection, contributing to the sternal dehiscence that warranted Robicsek repair during the index admission. The patient's prior history of smoking and diabetes mellitus, both known to contribute to impaired wound healing, likely increased his risk of these infections. Additionally, it was suspected that there may have been a degree of what is known as “post-pericardiotomy syndrome”. This is typically a febrile illness due to a pro-inflammatory reaction within the pleural and pericardial spaces following cardiac surgery. Due to the infection presenting with similar features, it may have been difficult to discern the exact etiology of the recurrent pleural effusions. As the index admission was not at our hospital, we cannot explain the exact details and thought processes related to clinical decisions made at that institution.

The patient initially underwent a Robicsek rewiring procedure after presenting with sternal dehiscence. The Robicsek technique, which involves a reinforced wiring pattern to stabilize the sternum, has been shown to improve outcomes compared to conventional sternal closure methods [12,13]. However, despite this intervention, the patient experienced recurrent sternal complications, including wire fracture and non-union. This underscores the complexity of managing these challenging cases and the need for a personalized approach based on the individual patient's anatomy, risk factors, and clinical presentation.

At our center, the patient underwent a comprehensive workup including CT imaging to assess the extent of sternal non-union and wire fracture. The decision was subsequently made to proceed with a complex sternal reconstruction involving the removal of the fractured sternal wires, rigid fixation with sternal plates, and application of amniotic membrane and platelet-rich plasma to promote wound healing. The use of rigid fixation with sternal plates has gained popularity in recent years, particularly for high-risk patients [14]. Plating provides greater stability compared to wire cerclage and may reduce the risk of postoperative complications. In a study by Snyder et al., sternal plating was associated with a lower rate of sternal wound complications compared to wire cerclage in high-risk patients undergoing cardiac surgery [11].

The incorporation of amniotic membrane and platelet-rich plasma in this case represents an innovative approach to promoting sternal wound healing. Amniotic membrane has been shown to have anti-inflammatory, antimicrobial, and pain-reducing properties, and may stimulate wound healing by promoting epithelialization and reducing scar formation [15,19]. The amniotic membrane is derived from the placental tissue of healthy, screened mothers, and is processed by MTF Biologics, a tissue bank that is highly experienced and regarded in the field of such biological technologies. The membranes contain angiogenic, anti-inflammatory, and antimicrobial factors, as well as intrinsic cytokines and matrix proteins that promote cell proliferation and tissue remodeling, as well as reduce fibrotic and scar tissue formation. Its bilayer structure additionally forms a physical and chemical barrier to infection, with its processing enabling the preservation of natural factors and their function [20]. Similarly, platelet-rich plasma contains growth factors and cytokines that may enhance soft tissue and bone healing [16]. While more research is needed to establish the efficacy of these biological therapies, their use in combination with rigid sternal fixation may offer a promising strategy for managing complex sternal wounds.

Postoperatively, the patient was managed with a multimodal pain management protocol emphasizing opioid reduction, which included the use of dronabinol. It has been shown that when this synthetic cannabinoid is administered perioperatively to patients undergoing cardiac surgery via a sternotomy, the total postoperative opioid requirement for adequate pain control is reduced by 40% [17]. This study also found a significantly greater improvement in cardiac function postoperatively in the group treated with dronabinol, which, while not necessarily relevant to the sternal recovery, is nonetheless an important outcome that will be of prognostic benefit to this patient. This approach is in line with recent efforts to minimize opioid use in cardiac surgical patients given the risks of dependency and other adverse effects.

To sum up, this report demonstrates the potential benefits of a multidisciplinary approach to managing complex sternal complications after cardiac surgery. The combination of sternal plating and novel wound healing adjuncts may offer a path forward in managing complex patients with recurrent sternal breakdown. However, further research is needed to establish the safety and efficacy of these approaches and develop evidence-based guidelines for managing these challenging cases. Emphasis should be placed on identifying high-risk patients preoperatively and implementing preventive strategies to reduce the incidence of sternal complications. Close postoperative surveillance and prompt intervention for early signs of sternal compromise are also essential to optimize outcomes in this vulnerable patient population.

## Conclusions

This case report highlights the complexities of managing recurrent sternal complications following CABG surgery and the potential benefits of a multifaceted approach to sternal reconstruction. The combination of rigid sternal fixation with titanium plates, application of amniotic membrane and platelet-rich plasma to promote wound healing, and a multimodal postoperative pain management protocol emphasizing opioid reduction represents an innovative strategy for addressing repeated sternal breakdown in high-risk patients. While further research is needed to establish the efficacy and safety of these approaches, this report underscores the importance of a personalized, multidisciplinary approach to managing these challenging cases, with an emphasis on identifying high-risk patients, implementing appropriate preventive measures, and prompt intervention when encountering early signs of sternal compromise.

## References

[1] Langer NB, Argenziano M (2016). Minimally invasive cardiovascular surgery: incisions and approaches. Methodist Debakey Cardiovasc J.

[2] Olbrecht VA, Barreiro CJ, Bonde PN, Williams JA, Baumgartner WA, Gott VL, Conte JV (2006). Clinical outcomes of noninfectious sternal dehiscence after median sternotomy. Ann Thorac Surg.

[3] Losanoff JE, Jones JW, Richman BW (2002). Primary closure of median sternotomy: techniques and principles. Cardiovasc Surg.

[4] Grmoljez PF, Barner HH, Willman VL, Kaiser GC (1975). Major complications of median sternotomy. Am J Surg.

[5] Schimmer C, Sommer SP, Bensch M, Bohrer T, Aleksic I, Leyh R (2008). Sternal closure techniques and postoperative sternal wound complications in elderly patients. Eur J Cardiothorac Surg.

[6] Schimmer C, Reents W, Berneder S (2008). Prevention of sternal dehiscence and infection in high-risk patients: a prospective randomized multicenter trial. Ann Thorac Surg.

[7] Matros E, Aranki SF, Bayer LR, McGurk S, Neuwalder J, Orgill DP (2010). Reduction in incidence of deep sternal wound infections: random or real?. J Thorac Cardiovasc Surg.

[8] Celik S, Kirbas A, Gurer O, Yildiz Y, Isik O (2011). Sternal dehiscence in patients with moderate and severe chronic obstructive pulmonary disease undergoing cardiac surgery: the value of supportive thorax vests. J Thorac Cardiovasc Surg.

[9] Dai C, Lu Z, Zhu H, Xue S, Lian F (2013). Bilateral internal mammary artery grafting and risk of sternal wound infection: evidence from observational studies. Ann Thorac Surg.

[10] Raja SG, Berg GA (2007). Should vacuum-assisted closure therapy be routinely used for management of deep sternal wound infection after cardiac surgery?. Interact Cardiovasc Thorac Surg.

[11] Snyder CW, Graham LA, Byers RE, Holman WL (2009). Primary sternal plating to prevent sternal wound complications after cardiac surgery: early experience and patterns of failure. Interact Cardiovasc Thorac Surg.

[12] Robicsek F, Daugherty HK, Cook JW (1977). The prevention and treatment of sternum separation following open-heart surgery. J Thorac Cardiovasc Surg.

[13] Tavilla G, Van Son JAM, Verhagen AF, Lacquet LK (1991). Modified Robicsek technique for complicated sternal closure. Ann Thorac Surg.

[14] Fawzy H, Osei-Tutu K, Errett L, Latter D, Bonneau D, Musgrave M, Mahoney J (2011). Sternal plate fixation for sternal wound reconstruction: initial experience (retrospective study). J Cardiothorac Surg.

[15] Schmiedova I, Dembickaja A, Kiselakova L, Nowakova B, Slama P (2021). Using of amniotic membrane derivatives for the treatment of chronic wounds. Membranes (Basel).

[16] Chicharro-Alcántara D, Rubio-Zaragoza M, Damiá-Giménez E, Carrillo-Poveda JM, Cuervo-Serrato B, Peláez-Gorrea P, Sopena-Juncosa JJ (2018). Platelet rich plasma: new insights for cutaneous wound healing management. J Funct Biomater.

[17] Kumar U, Macko AR, Kang N, Darian NG, Salek FO, Khalpey Z (2024). Perioperative cannabinoids significantly reduce postoperative opioid requirements in patients undergoing coronary artery bypass graft surgery. Cureus.

[18] Greenhalgh DG (2003). Wound healing and diabetes mellitus. Clin Plast Surg.

[19] Dolivo D, Xie P, Hou C, Phipps A, Mustoe T, Hong S, Galiano R (2021). A dehydrated, aseptically-processed human amnion/chorion allograft accelerates healing in a delayed murine excisional wound model. Exp Cell Res.

[20] Steed DL, Trumpower C, Duffy D, Smith C, Marshall V, Rupp R, Robson M (2008). Amnion-derived cellular cytokine solution: a physiological combination of cytokines for wound healing. Eplasty.

